# A SWOT Analysis of the Field of Virtual Reality for Firefighter Training

**DOI:** 10.3389/frobt.2019.00101

**Published:** 2019-10-16

**Authors:** Hendrik Engelbrecht, Robert W. Lindeman, Simon Hoermann

**Affiliations:** ^1^HIT Lab NZ, College of Engineering, University of Canterbury, Christchurch, New Zealand; ^2^School of Product Design, College of Engineering, University of Canterbury, Christchurch, New Zealand

**Keywords:** virtual reality, human factors, firefighting, training, SWOT

## Abstract

Virtual reality (VR) research has gone through rapid advances and the technology has established itself as a valuable training tool in many domains. While research in the field of emergency response, and more specifically in the field of firefighting, is still catching up, the future potential of VR technology for training is promising. This paper uses the SWOT framework to analyse the strengths, weaknesses, opportunities, and threats immersive VR technology faces in the field of firefighter training. While using VR for training is cost-effective, safe to use and provides the ability to prepare trainees with a large variety of high fidelity training environments, the lack in specialization of the applications for the fire-service sector and issues with technology acceptance and limitations need to be addressed. Looking to current research, there are promising findings that might be directly transferable, creating affective, and multi-sensory experiences for more effective mental and physical training of firefighters in the future. More research is needed to establish methods of skills transfer from VR to real life scenarios and to evaluate the potential risk of frequent training in engaging and physiologically stimulating virtual environments.

## 1. Introduction

Virtual reality (VR) has come a long way from an exorbitantly expensive niche technology to an affordable consumer product used for entertainment and professional purposes. Looking at *Gartner's Hype Cycle* for the year of 2017, we can observe VR technology being on it's way to the plateau of productivity ^1^. It was consequently even removed from *Gartner's* evaluation this year as it was deemed a mature technology, no longer falling into the category of emerging technologies^2^. But determining the *maturity* of VR is a rather multifaceted problem. Due to the broad variety of VR applications being used for entertainment, research, and training purposes, VR needs to be investigated from each of these angles separately to determine its potential and relative maturity.

The current investigation will look into the potential of *immersive* VR exclusively. While the term VR has been used extensively in research for immersive and non-immersive simulation based training, the researchers argue that the immersive properties of head mounted displays (HMDs) and CAVE systems (Cruz-Neira et al., 1993) enable training that occupies a special positions compared to general simulation training. The term VR will henceforth be used as synonymous with immersive VR for the remainder of this paper (unless specified otherwise).

From entertainment, through education, to clinical purposes, VR has become a tremendously promising technology, with research efforts moving at a record pace over the past years (Anthes et al., 2016). Especially the clinical domain has seen a large variety of applications, with Rizzo and Koenig (2017) deeming VR *ready for primetime*. While other domains are still treading in the shadows of their clinical colleagues, the possible applications are promising and call for more thorough investigation into their possible future value.

VR permits the creation of infinitely large and complex training environments, which enables the training of scenarios that are difficult, or immensely resource intensive, if done in real life. This represents a good fit with training carried out in the emergency response domain and so it is no surprise that VR has received significant attention in this field (Hsu et al., 2013). Among the occupations in the emergency response domain, the job of firefighter has a unique status with regards to the skills needed and threats faced during everyday deployment. While there is an overlap between firefighters and other emergency response professions, firefighters face a larger amount of varied environmental threats. As summarized by Dunn in *Safety and Survival on the Fireground* (Dunn, 2015):

“*Firefighting is a high-risk, dangerous occupation. The major risks of firefighting are from explosion, collapse, falls, falling objects, rollover, flameover, flashover, backdrafts, fire, smoke, heat, disorientation, and electrocution. Unlike other dangerous occupations, firefighters work in an extremely dangerous environment, constantly threatened by death, and injury when performing lifesaving tasks.”*

From wildfires, chemical spills, search, and rescue during natural disasters such as earthquakes, urban firefighting in confined indoor spaces to aerial firefighting, the environments and threats faced vary immensely. VR presents the potential to enable a safe, immersive, and cost-effective way of training for high risk incidents in varied and complex environments. With the training needs of fire service employees being high and rising^3^, VR could potentially be a tool for preparing firefighters, physically as well as mentally, for real life incidents. As often found as a quote in fire departments, according to Goodson and Murnane (2008), the importance of training for firefighters can be seen in a single statement:

“*Train as if your life depended on it - it does.”*

For this paper, the *SWOT analysis* tool will be utilized to analyse the strengths, weaknesses, opportunities and threats being faced when applying VR for the training of firefighters. While the SWOT framework was originally conceived as a way of analyzing market forces impacting the standing of companies, it has been utilized successfully in academia to analyze the application of emerging technologies in a specific field (see for example Rizzo and Kim, 2005). The following sections will describe and discuss the strengths and weaknesses of the VR technology for firefighter training as evidenced by research in the field of emergency response, firefighting, and other domains. The second half will contrast these with possible future opportunities and threats that emerge as logical extensions of developments in the VR space (Table 1).

**Table 1:** Overview of SWOT findings.

**Strengths**	**Weaknesses**
- Cost effectiveness - Complex and varied training scenarios - High ecological validity - Increased safety for high risk training - Trainee engagement - Data recording	- Lack of specialization and testing of systems - Immaturity of technology - Technology barrier- Lack of multi-user fidelity
**Opportunities**	**Threats**
- System engineering progress - Transfer of findings from other domains - Increase in physical fidelity - Increase resilience against adverse effects	- Uncertain skill transfer - Worsening of overall net-effects of training - Adverse effects of habituation - Adverse effects of engagement stimulation

This SWOT analysis can only provide the state of technology currently released (or being developed) in the field of firefighter training and related fields. With the fast pace of technology developments in the VR space opportunities might either become reality or evidence will be established to disprove their respective utility. In the same way, weaknesses may become future opportunities due to the advances in technology and human factors developments. This assessment aims to provide an entry-point to inquiries in the near future. Ultimately, the obsolesce of this work will be a sign of progress and be preserved as a snapshot of our period.

## 2. Strengths

### 2.1. Cost Effectiveness

**Figure 1:**
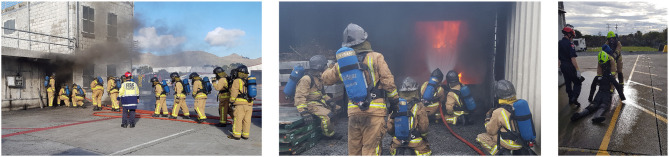
Breathing apparatus training in live fire environment for New Zealand firefighters.

According to the annual report of the *New Zealand Fire Service*, a total of 2,786 courses were carried out in the fiscal year 2018, which constitutes a throughput of 21,608 people. In 2018 alone, a total of 16 structures were acquired for live structure fire training, with 280 volunteer participants taking part in the exercises^4^ (see, for example, Figure 1). Looking at these numbers, it is easy to estimate that training costs make up a large part of the annual budget of any fire department around the world. Budget is one of the biggest threats to adequate training and comes under threat quickly in times of financial crises (Buckman, 2005). Obtaining a reusable structure for live fire training can easily cost up to one million US dollars^5^, making it difficult for smaller fire departments to administer live fire training to their employees. The large variation in training scenarios further increases the cost of training, since training grounds, equipment, training processes and teachers all need to be especially adapted for the different circumstances. Training the handling and removal of hazardous substances (e.g., in the occurrence of a chemical leak) requires entirely different routines, equipment and vehicles than wildfire exercises, which are largely dependant on the coordination of resources for aerial firefighting.

VR can, in theory, handle all these training scenarios in the same location. While the initial cost for development of the training simulations and the purchase of the equipment may be high, this can arguably be offset by the relatively cheap adaptation of the content for different scenarios, the lower cost regarding upkeep of the training equipment, the portability of the equipment for saving transport cost of trainees to training sites and the relatively modest space needed for the training itself. With the market offering a wide range of VR systems, the weighing of costs vs. quality can be made from HMDs (utilizing mobile phones) all the way to elaborate *CAVE* systems (Cruz-Neira et al., 1993). Training or education in VR can be done with relatively little funds if lower fidelity is acceptable, see for example in the general education domain [(Brown and Green, 2016; Vishwanath et al., 2017)].

Equipping every fire department with the potential to train their firefighters in a wide range of environments and scenarios at a fraction of the cost could greatly increase the general preparedness throughout departments all over the world. This can be seen as a democratization of what used to only be feasible for a small subset of fire services around the world—affordable and immediate training in immersive environments almost regardless of the size or budget of the department.

### 2.2. Complex and Varied Training Scenarios

Utilizing VR technology for training enables trainees to experience a wide variety of training scenarios in a single session. In the field of VR training for firefighting research efforts have been made to build systems simulating wildfire-fighting for ground troops (Hoang et al., 2010), wildfire-fighting for aerial support (Clifford et al., 2018b), urban firefighting (Xu et al., 2014), and incident leadership (Cha et al., 2012). The environments being trained in vary widely depending on the job category of the firefighter and the incident being trained. The simulation environment of a fire on a ship (Tao et al., 2017), an offshore platform (Koo et al., 2017) or even a space-ship (Ruff et al., 2005) all vary widely in the procedures trained and the dangers being faced. The complexity of the training can range widely from the skill training of relatively fine motor skills for specific equipment to large scale live training operations that require the training of communication and coordination skills.

Training of skills only applicable to situations with a high degree of complexity is a very expensive and difficult undertaking. For example, the repeated training of fire evacuation procedures (Sharma et al., 2017) or learning to communicate effectively between different emergency response services in crisis scenarios (Molka-Danielsen et al., 2018) would be immensely complex and expensive to coordinate in real life due to the large amount of equipment and personnel needed for these scenarios.

Having the control over what is being trained in a VR simulation for each trainee individually further makes it easy to train different categories of personnel in a single unified scenario, without having to drain the personnel resource of the department for each aspect being trained. While the VR simulation can be used to train the hands on procedures of fighting a fire inside a building, the commander can utilize the same simulation to train his skills at deploying and coordinating resources. While real life training exercises have limited repeatability, having control over all aspects makes VR training highly adaptable and repeatable. During training the same aspects of a scenario can be set up in the exact same way to improve subsets of skills needed. Being able to collect data during a highly standardized training process also enables not only the exact identification of trainee weaknesses, but also direct feedback to correct those weaknesses during training.

Training of firefighters is complex and varied, which creates bottlenecks for the coverage of all trainees on a frequent basis. VR offers a solution to that bottleneck by being adaptable to almost the entirety of the fire service workforce.

### 2.3. High Ecological Validity

VR as a training tool has been receiving a lot of attention due to its immersive properties. Gains from training in fully immersive environments have been shown to outweigh the gains obtained in monitor bound training, for example in the field of medicine (Coulter et al., 2007) or aircraft construction (Vora et al., 2002). Higher visual immersion, using a HMD, increases the overall presence in the virtual environment, which can improve training performance (Stevens and Kincaid, 2015). Although VR for training purposes needs to be balanced according to its possible advantages and disadvantages, presence does undoubtedly have an important role in learning (Pantelidis, 2010; Mikropoulos and Natsis, 2011).

In the field of firefighter training, using immersive technology can be an especially valuable tool since environmental threats comprise a large part of the threats encountered. Creating a training environment that accurately portrays these threats, and stimulates the corresponding psychological responses, can prepare trainees for the conditions of the real task by immersing them in it. Psychological stress, and the accompanying physiological responses, have been successfully elicited throughout a variety of studies (see for example Kotlyar et al., 2008; Jönsson et al., 2010), often not falling short of their real life counterparts. Stress elicited by environmental conditions has a direct relationship with performance (Welford, 1973), which makes the elicitation of it in training scenarios all the more important.

Affectively demanding environments train the user to deal with the same conditions during real life training and later execution of the actual task (Wilfred et al., 2004). The threat of a house engulfed in flames can only be portrayed with very limited fidelity and trainees experience limited visual and physical immersion using monitor based simulations. The stress and anxiety experienced, as brought upon by the immersion and presence in the VR simulation, prepares firefighters for the adverse conditions faced during real deployment.

Being able to approximate the real world conditions by evoking nearly identical physiological and psychological responses during training means high ecological validity that is of great value to research and training purposes.

### 2.4. Increased Safety for High Risk Training

It is not difficult to imagine the possible dangers associated with live fire exercises, despite a high amount of precaution taken to increase safety. Having firefighters train in a high risk scenario always carries a danger that is only outweighed by the benefit this kind of training brings to the trainees.

In 2017, there were a total of 8,380 injuries and ten deaths caused by training accidents in the United States alone (Evarts and Molis, 2018; Fathy et al., 2018). As elaborated above, VR enables work environments with high ecological validity that may be able to replace the live fire exercises entirely in the future. Even if VR does not entirely replace traditional training exercises, it offers trainees the possibility to receive additional training for high risk scenarios without encountering any risk associated to it. Despite concerns regarding negative side-effects experienced by some, e.g., the experience of cybersickness, the potential benefits of VR tech outweigh the potential risks encountered during usage for training purposes (Nichols and Patel, 2002).

While replacing real life fire exercises with VR training completely is still not a feasible solution in the near future, supplementing real life training with additional VR training, while slowly increasing its share of training time, will over time increase the overall safety of firefighter training without affecting training effectiveness.

### 2.5. Trainee Engagement

Adequate training requires long term commitment and repetition in order to achieve desired results. Procedures and protocols need to be carried out over and over to internalize the mental schemas and motor skills required for the real life scenario. Training adherence is a key factor for success in the firefighting domain. There needs to be a constant reinforcement of the techniques learned to internalize the skills and procedures (Goodson and Murnane, 2008).

The rewarding facets during real life training include feedback being given by instructors and intrinsic factors associated with self-improvement and efficacy desires, which can relate to team- as well as individual performance. Furthermore, outcome goals, such as increased social unity among trainees and career advancement, can be motivating factors. Using simulation training technology enables the creation of extrinsic reward and feedback systems to further improve engagement. For example, the use of real-time feedback to relate skill execution to performance can provide a challenge that foreshadows real-life consequences, as was done with the inclusion of a health system in Backlund et al. (2007). Data collection can thereby not only be used as a means to *post-hoc* analysis, but can directly be appropriated for feedback systems that display relevant metrics for improvement and engagement.

The improvement of engagement will ultimately lead to better training adherence and thereby to better training outcomes. Situated learning, as enabled by VR technology, facilitates learning (Dede, 2009). Interacting actively with the training content in a virtual environment increases the motivation of trainees to adhere to training (Mantovani et al., 2003).

The engaging properties of serious games are a promising way of increasing training adherence and effectiveness. In a literature review of serious games and simulations used for fire service training (Williams-Bell et al., 2015) conclude that while the benefits of serious games have not been fully utilized as of yet, it can greatly benefit firefighter education. For the application of game environments for training in VR, there have been promising findings showing successful implementation of urban firefighting training. The SIDH simulator (Backlund et al., 2007) lets trainees practice search and rescue tasks through a series of 13 levels. Appropriate behavior, such as assuming a crouching stance to avoid smoke and heat, is fostered by appropriate feedback mechanisms during the game and scoring at the end of each level.

Increasing training adherence through engaging and immersive training environments is a good step toward more training and ultimately a better prepared firefighting population.

### 2.6. Data Recording

Although not specific to VR simulation training, the recording of user data during training is a strength that extends to VR. Reflective thinking and reconsideration of plans in highly dynamic environments is a valuable skill for jobs in the emergency response domain, due to the nature of the highly variable and stressful environments these jobs are performed in. This makes experiential learning approaches, such as live-fire training and role-playing, an important part of training. Utilizing simulation training, scenarios can be structured to not only represent highly dynamic scenarios, but further can utilize user data to adapt the experience. Tutors can be replaced by intelligent tutoring systems (ITS) that adapt to decisions in real-time. Although ITS systems can differ greatly in how they adapt to trainees, they can be nearly as effective human tutors (VanLehn, 2011). Another cornerstone for adaptive learning in simulations and real training is the after action review (AAR), which incentivizes trainees to reflect on decisions based on instructor feedback and their self-assessment. Utilizing data collected during training enables objective and automated measures of performance that can be fed back and visualized during the AAR (see, for example, Jenvald and Morin, 2004). The automation of objectively measuring performance related data together with the assistance of trainers for reflection can lead to better learning outcomes for emergency response personnel (Hanoun and Nahavandi, 2018).

Where VR differentiates itself from monitor bound simulation training is in the collection of data relevant to body posture and gaze. With arguably higher interaction fidelity, VR training can record not only decision making metrics, but assess performance on motor skills and relative attention paid to environmental stimuli. As Backlund et al. (2007) showed in their study, body posture feedback can be applied to train firefighters to assume a crouching position when entering a building with an active fire. Gaze can be used to track performance in terms of situational awareness or even to obtain relevant data from experts to improve gaze patterns for novice trainees. For example, Wilson et al. (2010) were able to evaluate what differentiates novice from experienced surgeons by analyzing their gaze in the VR simulator.

The development of adaptive systems utilizing VR training data is increasing (Vaughan et al., 2016). This adaptation can take many forms, but at its core data provides the crucial input that differentiates VR from traditional forms of training. The possibility to alter scenarios and difficulty in accordance with individual needs, provide immediate real-time feedback and measure performance objectively for AAR reflections enhances the effectiveness of experiential learning.

## 3. Weaknesses

### 3.1. Lack of Specialization and Testing of Systems

VR hardware has become affordable, even for consumers, and with that a lot of research and commercial efforts have sprung up to use VR for training of emergency response teams, see for example the *LUDUS* (LUDUS, 2019) or *NAFFCO ARFF* (Sense-R, 2019). The LUDUS simulator offers customizable scenarios for ports, airports, forests and urban firefighting which let the user train strategic decision making processes from a first person point-of-view. The NAFFCO ARFF system, on the other hand, trains the actual task execution of extinguishing a plane fire while utilizing hardware that mimics real life controls of a firefighting vehicle.

While the fidelity and ecological validity achieved with current hardware is commendable, without the use of specialized hardware, such as the input methods of the NAFFCO AR (Sense-R, 2019), there is a gap as to what the technology could achieve for any given profession. There is an undeniable skill-overlap between the different emergency response professions, especially when only the cognitive skills are considered. People working in the emergency response domain need to be able to work under time pressure, while retaining high situational awareness in order to make correct decisions. However, this does not apply to the entire range of skills and demands faced by emergency response teams. As opposed to, for example, police officers, trainees of the fire service are encountering environmental threats with high intensity more regularly and are tasked with containing them.

Similarly, the actual physical skills needed are often unique to the fire service, with a wide range of specialized equipment to use and dangers to face. Addressing these unique aspects of firefighting in its many variations demands the development of specialized hardware and software to offer optimal training in VR. The researchers argue that the skills trained in generic systems for emergency response have only limited applicability to trainees in the fire service. For example, while there is an abundance of research into the behavior of civilians in fire emergency VR simulations, assessing the potential of training spatial navigation (Vilar et al., 2014), looking at psychological stress during navigation behavior (Meng and Zhang, 2014), or the impact of social information on way-finding (Kinateder et al., 2014), there is very little work being done to see how these findings could apply to firefighters entering the building for search and rescue tasks.

This shortcoming further extends to the input devices used for current VR simulations, where standard motion controllers, as they are shipped with the corresponding HMD, are used in most cases. The adaptation of interaction design patterns with specialized hardware could have a tangible effect on training. One example that has presented a considerable challenge for researchers is that of locomotion in VR, with traditional input methods, i.e., a joystick to move the player in the simulation, leading to cybersickness which is believed to be caused by the sensory conflict between of the visual input given to the user and the motion performed (Rebenitsch and Owen, 2016). While there are a lot of efforts to mitigate the problem, e.g., by mapping controller input in a more natural way to simulate a walking motion (Sarupuri et al., 2017) or using omnidirectional treadmills (Souman et al., 2011), as of the writing of this paper there is no system that allows natural movement in large VR spaces (Nilsson et al., 2018). The closest approximation to natural locomotion is in the application of walk-in-place techniques, which has not yet developed far enough to provide a perceptually natural walking motion (Nilsson et al., 2016).

Natural motion mapping in VR has been linked to increased spatial presence (Skalski et al., 2011; Seibert and Shafer, 2018), which means that the usage of general purpose input devices could hinder presence and subsequently training effectiveness. Breaking down the difference between the mental script of the real situation and that emerging from the simulation, by using natural interaction methods, ultimately aids the skill transfer from training to the real event (Skalski et al., 2002).

Another roadblock is the lack of evaluation being put forward by researchers developing specialized applications. While evaluation does often take place on a system level (see for example Cha et al., 2012; Yuan et al., 2012), the actual training effects for fire service personnel are not within the scope of many investigations. Studies investigating human factors do exist, but are few (Clifford et al., 2018a; Puel, 2018) or have limited applicability to the standard of VR simulations today as hardware and software are of considerable age (Rosenblum et al., 1996; Bliss et al., 1997).

Before any value can be ascribed to VR technology for firefighter training, the gap to actual application of the technology needs to be closed with methodologically sound user studies. This can be achieved by using professionals in the field, deploying specialized applications that take the unique attributes of the firefighting population into account and utilizing hardware that mimics natural inputs as closely as possible.

### 3.2. Immaturity of Technology

Despite VR having been taken off *Gartner's Hype Cycle*^6^, there are still a great number of limitations to overcome. Strides have been made in improving frame-rate, tracking, field of view, refresh rate, latency, and resolution over the past years (Anthes et al., 2016), but the road to photo-realistic VR is still long.

With presence being highly dependent on tracking technology and wide field of view (Cummings and Bailenson, 2016), both need to be improved substantially to achieve optimal training outcomes. Visual artifacts, such as the infamous screen door effect, i.e., the visibility of pixel borders due to the close proximity of the display to the user's eyes, have an effect on the level of visual fidelity being experienced. While higher pixel density can reduce the problem, current HMDs are still experiencing the issue (Desai et al., 2014). Low latency is important to create not only a more pleasurable immersive experience, but also to heighten presence (Meehan et al., 2003). Latency, especially for wireless VR training, is still a current issue that needs more research (Elbamby et al., 2018).

Anthes et al. (2016) describe the current state of VR developments as *fast and promising*, but list numerous problems that are still difficult to tackle, like user representation in HMDs, cybersickness, and distance perception in virtual environments. In a study by Clifford et al. (2018b) aerial firefighters were asked to state their preference after testing the same simulation on (1) a standard monitor, (2) using a VR HMD, or (3) using a simulation “pit” outfitted with three projected cockpit windows. Although preference was given to the HMD with regards to immersion, the issues with cybersickness experienced by trainees made them prefer the monitor. Considering widespread implementation of the VR training above, the issue of cybersickness alone could threaten overall training effects due to technical limitations.

For VR training to actually replace or supplement real life exercises in the firefighting domain, the technological shortcomings need to be ironed out. Maladapted training for low risk occupations results, in a worst case scenario, in worse performance and productivity. In the fire service, adequate training can mean the difference between life an death, which elevates the standard for what the technology needs to provide to be considered as a viable widespread training standard.

### 3.3. Technology Barrier

Acceptance or dismissal of new technologies can be a critical factor (for researchers and industry) for the development of novel and innovative systems. According to the technology acceptance model by Venkatesh and Davis (2000), subjective norms, perceived usefulness and perceived ease of use determine the successful adoption of new technologies by consumers. The model has been applied to a variety of VR applications to highlight the dependency of training results on the acceptance by trainees in the medical domain (see for example Huang et al., 2016). Virtual spaces for training need to be easy to use and provide an enjoyable experience to the learners in order to be perceived as useful (Tokel and İsler, 2015). With the issues highlighted earlier, concerning the immaturity of interaction design techniques and user experience, VR technology faces more barriers than non-immersive simulations.

As has been shown by Engelbrecht et al. (2019), new technology in the emergency response sector needs careful adaptations to work routines currently performed in order to be accepted by professionals. Especially among older generations within the workforce, the resistance against new technology implementations in the institution can be a roadblock toward adoption (Morris and Venkatesh, 2000).

In the domain of firefighting, the issue of technology acceptance is not new either, and professionals can be rather cautious with their expectations of the potential of innovative technology (Weidinger et al., 2018). The issue of masculinity as a disruptive force is well documented in the field and does create resistance toward organizational change (Thurnell-Read and Parker, 2008). The idea of technology assistance being seen as a threat to the skill-set required can be observed for even the most rudimentary technology in the past. Nowadays seen as a standard piece of equipment, the breathing apparatus was met with a lot of resistance due to the perceived loss of skills usually needed without one (Baigent, 2001).

The firefighter instructors play a key role in implementing new technologies for the trainees and the bridge to their traditional ways of working must be created to ensure adaptation (Roberson-Moore, 2018). The responsibility on part of the instructors, having to teach possibly life saving skills adequately, is not to be underestimated and therefore convincing teachers and upper management is a key part of the process before even the individual can be considered.

Seeing how dependent firefighters are on thorough and intensive training to mitigate the risk of personal harm, how diverse the landscape of firefighter training is and how novel VR technology is to the domain, trusting new technology to provide adequate training is an understandably difficult ask.

### 3.4. Lack of Multi-User Fidelity

Firefighting is a team-effort. Regardless if fighting a wildfire or urban fire, a firefighter will never enter a fire-attack scenario alone. Many tasks further even require the presence of two firefighters, like door entry techniques or search and rescue evacuations (see Figure 1), which creates a gap for the skill transfer from solitary VR simulations. As mentioned in an earlier section, virtual agents can be used as instructors and the same has been has been applied to using virtual agents as firefighters to train commanders (Puel, 2018). Using virtual agents as training partners for firefighting simulations has been explored as well (see, for example, Lee et al., 2010), but ultimately fails to reach the interaction fidelity needed for training of cooperative tasks beyond decision making. Due to the limited visibility, firefighters make heavy use of touch to not only orient themselves, but also to communicate with their partners.

For example, the evacuation of a victim (see Figure 1) in a search and rescue scenario, requires one firefighter to guide the other by physically pulling him from behind. The adaptation of this task into a simulation can't train the physical cooperation needed for mirroring this task with appropriate haptic fidelity. The same applies to spatial orientation using the walls and objects within a room in an active fire scenario. Due to almost zero visibility, in the case of a burning building interior, constant communication, and touch is used to ascertain what has been identified regarding the room type and layout, as well as the current location of other firefighters. Mimicking this in VR currently would require a replica of the exact room due to the lack of advanced enough haptics and accurate spatial sound, which in turn eliminates the advantage of low cost VR training.

## 4. Opportunities

### 4.1. System Engineering Progress

As elaborated earlier, the human factors perspective is still missing a great deal of studies to build a conclusive picture of VR training in the firefighting domain. Despite this, a lot of work has been done on the system engineering level of applications that may one day be adapted for actual implementations in the field.

The quick spread of fire represents a serious danger to firefighters. Escape routes can be cut off and sudden flame-overs can trap firefighters within seconds (Dunn, 2015). The general behavior and spread of fire in VR training needs to be realistic in order to heighten the physical and visual fidelity important to the accurate learning of skills. The incorrect simulation of fire would lead to a completely inaccurate training on how to extinguish it and potentially have life threatening consequences. Fire spread in different environments needs to consider a multitude of different factors influencing it, which has resulted in a large body of work covering, among others, the spread of fire in industrial facilities (Dedov et al., 2017), earthquake emergencies (Lu et al., 2017) and forest fires (Yun et al., 2011; Huang et al., 2016). A system developed by Moreno et al. (2014) does not only simulate spread, taking into account factors such as wind and flying embers, but also approximates the use of firebreaks and extinguishing agents on the spread of fire.

The same applies to the realistic simulation of smoke to accurately reflect the visual obstruction and volumetric fill of it. Spatial navigation skills applied during limited visibility conditions are important especially for urban firefighting. An inadequate portrayal of reduced visibility in VR due to smoke could provide inadequate training. Work on simulating smoke accurately has been carried out to build systems for training emergency evacuations (Ren et al., 2008), which rely heavily on spatial navigation skills. Many of the VR systems engineered for simulating fire spread are also naturally covering the simulation of realistic smoke, which creates a large basis to work with when creating VR training simulations in the future.

Lastly, there is a lot of work addressing the accurate simulation of crowd and individual behaviors in panic situations. In line with the need for accurate smoke and fire spread in different scenarios, the realistic depiction of civilian behavior in a fire could help to increase the fidelity of training. Especially being able to accurately model and simulate evacuation behaviors for subway stations (Pelechano and Malkawi, 2008) and high rise buildings (Ronchi and Nilsson, 2013) could train firefighters in scenarios which involve the anticipation of the behavior of large crowds in confined spaces reacting to a fire.

While these incremental steps in the engineering of algorithms and overall systems has not yet been applied in practice in many cases, the progress in this domain has great applicability to the future of VR firefighting training systems.

### 4.2. Transfer of Findings From Other Domains

VR as a tool for training has been investigated in a large variety of domains. Although not all findings might be directly transferable to the field of the fire service, findings from other domains might give a good indication for the opportunities VR brings to firefighter training in the future.

In the medical domain there have been fruitful efforts to train surgeons with non-immersive VR technology for laparoscopic surgery. Over a wide range of studies, the training proved to be more effective than video training and on-par with other training methods used (Alaker et al., 2016). While the evidence found is very domain specific, the usage of VR for fine motor training tasks could be directly transferred to, for example, the training with portable fire extinguishers. The same can be hypothesized about more rough motor skill training, e.g., the handling of large equipment such as fire hoses, ladders, or vehicles. Firefighters need to train with a variety of equipment, which all demand different procedures for correct usage. Using VR as a means to train the motor skills and procedures necessary, ideally using natural input interfaces, would enable the training on equipment that does not even have to be in the possession of the department.

VR is a powerful tool to enable perspective taking and to create empathy. In VR storytelling research empathy has been successfully elicited and been related to embodied cognition (Shin, 2018), which enables the user to feel like their virtual counterpart (Kilteni et al., 2012). In high risk situations, the interaction with the victims and people at risk of injury can potentially lead to catastrophic outcomes if panic breaks out or procedures are not followed correctly. Utilizing VR to create empathy for training firefighters opens up a new way to train interpersonal contact in these situations. Taking the perspective of, for example, a person trapped in a burning building could improve the interpersonal skills needed when dealing with people involved in an emergency scenario.

The demands of the work of emergency response teams further encompasses the interaction with colleagues in high risk scenarios. While still preliminary, simulating interpersonal factors through training scenarios in VR, with automatic agents controlled by artificial intelligence, has been done successfully (Sharma et al., 2017). Similarly, sharing a virtual space with colleagues can be beneficial for education and training (Greenwald et al., 2017). Being virtually co-located, while feeling present in the virtual environment, opens up new opportunities for training that are distinct from non-immersive networked simulations.

It is difficult to distill all the possibilities into a handful of findings due to the vastness of the field. For the field of VR firefighting training the transfer of findings on motor skills training, embodied cognition, and shared virtual spaces are some of the most promising for near-future developments.

### 4.3. Increase in Physical Fidelity

What VR technology can achieve today in terms of simulation fidelity is far from where the field was a decade ago. Computing power will likely not receive the same increase it has seen over the past decades and with that the further increase in visual, and ultimately physical, fidelity of VR systems will need to be aided by other improvements. This is not to say that there can not be improvements in visual fidelity. Optimizing the pipeline of displaying VR content to the user can still optimize the computing resources greatly and thereby make it possible to improve the visual fidelity by e.g., optimizing how the content is rendered. One example of this would be the fairly recent discovery and implementation of foveated rendering, which helps to cut down on elements that need to be rendered by tracking the gaze of the user (Patney et al., 2016).

One has not to necessarily only look at improvements in visual aspects when it comes to increasing fidelity. The eyes are only one pathway in strengthening the physical fidelity of the virtual environment and consequently the immersion of users. Including other senses as a mediating factor in creating highly immersive environments is a promising opportunity. Other fields have made use of haptics from simple vibrations for collision feedback applied to wrists and temples of the hands (Nukarinen et al., 2018) through haptic gloves with resistance for multiple fingers (Blake and Gurocak, 2009) to feedback provided on multiple body parts (Konishi et al., 2016). This even enables the simulation of the physical presence of a virtually co-located colleague not physically present in the training space (Swapp et al., 2006). With future developments in haptic feedback, the physical characteristics of a training environment can be improved dramatically to more adequately prepare firefighters for the demands of the corresponding real environment. The effects of heat radiation in training scenarios has been looked at in the context of VR training for fire emergency scenarios. The participants in Shaw et al. (2019) reported to be feeling more like actually *being in a fire* in the multi-sensory condition, which led to more natural actions during the experiment.

Haptics is only one promising area that could greatly improve the immersion of VR training. Research into olfactory stimulation in the domain of medical simulation training has been promising with the use of smells benefiting training outcomes due to better recall of knowledge, improved physical fidelity and the desensitization to malodours (Kent et al., 2016). The use of olfactory stimulation in VR has been shown to have similar psychological effects as traditional stimuli, e.g., enabling the elicitation of food cravings (Tuanquin et al., 2018) or alcohol cravings (Bordnick et al., 2008). Little research has been done using olfactory stimulation in the VR firefighting domain, with some of the few studies stemming from research being done with very early VR hardware (Cater, 1994). Looking to the work by Feng et al. (2016), using a combination of multisensory cues increases the user preference, with more cues relating to a higher preference. This highlights the second opportunity of multisensory VR simulations as not only a tool for higher physical fidelity, but also higher engagement possibly leading to increased training adherence.

It is easy to pass off a potential threat during virtual training due to the non-threatening characteristics of the training environment. Multi-sensory VR training enables trainees not only to see the threat, but to feel it. There are a wide range of skills needed in firefighting that rely heavily on sensory input. The smell of leaking gas, the change in wind direction or the heat radiation outside a door are all life saving sensory cues that are necessary for adequate risk assessment and need to be a part of training. Furthermore, utilizing multi-sensory stimulation to full effect during VR training could increase the immersion of trainees and therefore make skills more easily transferable between VR and real life.

### 4.4. Increase Resilience Against Adverse Effects

Mental preparedness is important for firefighters to reduce the possible experienced emotional and psychological distress (Goodson and Murnane, 2008). Helping and interacting with the victims in emergency situations can be a traumatic experience that requires mental hardiness to prevent adverse effects. With alarmingly high suicide rates compared to the general population (Antonellis and Thompson, 2012; Henderson et al., 2016) and PTSD rates significantly higher than many other professions (Corneil et al., 1999), firefighters are a unique population at great risk of adverse mental effects stemming from their work environment.

With the possibility of creating high physical fidelity and immersion in VR simulations, we can hypothesize about the opportunity of VR as a tool to increase mental preparedness by exposing trainees to stressors in a safe environment. In the healthcare domain, VR has proven to be a promising tool to treat patients with anxiety disorders and phobias (Parsons and Rizzo, 2008; Powers and Emmelkamp, 2008). Further, there is evidence to suggest that VR exposure therapy is as efficacious as regular exposure therapy (Gonçalves et al., 2012). As opposed to traditional training, training simulations enable the exact control of the environment during training. Stressors can be introduced, controlled, and reduced based on the need of the trainee. Combining this with the use of physiological measurements, the possibility of precisely adaptive training is possible for better training outcomes by keeping physiological arousal at the optimal level of performance (Anderson, 1990).

Taking the strength of the findings in exposure therapy, we can hypothesize about the immense impact VR could have on reducing physiological responses through exposure for firefighters. Using some of the same mechanism of VR as an affective environment, it can be applied not only as a tool to treat but to prevent. The environment in which a firefighter operates often requires the regulation of emotional, physiological and psychological responses as a result of the stressful work environment. Gradual and repeated exposure to stressors during training, to optimize performance, has been long used in high risk occupations. This has been formalized with the term stress inoculation training (SIT) as a treatment and preventative approach (Meichenbaum, 2017). SIT is a well-established tool for military personnel (Hourani et al., 2011), with promising results stemming from the SIT VR training of combat medics (Wiederhold and Wiederhold, 2004) and soldiers (Stetz et al., 2007). Although there is undoubtedly a difference in the type and valence of threats between soldiers and firefighters, the general principles should be applicable to the fire service as well. Findings from the clinical domain further support this notion with strong evidence for the efficacy of VR stress resilience training (Rizzo et al., 2012, 2013).

The proposed approaches for increasing the resilience of firefighters include not only the exposure to stressful stimuli, but also the teaching of cognitive skills to increase self-efficacy and thinking habits that reduce anxiety (Deppa, 2015). Utilizing VR tech for the training of cognitive skills has the advantage of the trainee being placed into the situation where the skill needs to be applied later.

VR training provides tremendous opportunities to improve the mental health of the firefighting population. Taking the well-established evidence in the military and medical domains, there is a lot of promise in the future transfer of these findings specifically for firefighters.

## 5. Threats

### 5.1. Uncertain Skill Transfer

While the usage of VR for the training of firefighters has resulted in some promising findings for commander training (Cohen-Hatton and Honey, 2015), aerial attack coordination (Clifford et al., 2018b), and urban firefighting (Bliss et al., 1997), there is still uncertainty whether the skills learned and trained in VR transfer to real situations.

There has been quite some success in the medical domain in the transfer of motor skills from non-immersive VR simulations to the operating room (Torkington et al., 2001; Gallagher et al., 2013; Yiannakopoulou et al., 2015). Though these findings are well documented, they are mostly looking at the transfer between high interaction fidelity simulations for the training of fine motor skills. Arguably the most studied example is that of laparoscopic tasks for surgery (Larsen et al., 2012). All interactions with the actual patient are mediated by the laparoscopic instruments and can thereby easily be simulated in a virtual environment given the right input devices (constituting a natural interface). For other motor skills training the findings are more mixed.

Gavish et al. (2015) found no significant differences in performance between non-immersive VR and video instructions in a maintenance and assembly task, while the training time required was longer using non-immersive VR training. With the added complexity of executing VR training, as compared to watching an instructional video, the VR training benefit needs to clearly outweigh its cost. Also using an assembly task, Sportillo et al. (2015) did find that participants using a VR trainer did get better over time on the virtual task, but this did not translate to better performance on the real world task.

Firefighting requires a very broad range of mental and physical skills with a multitude of equipment deployed for specific purposes. A lot of research is needed to assess whether current (and near future) hardware and systems actually can provide enough skill transfer to replace simple to apply traditional learning methods. The worst case scenario would be the implementation of unproven VR training, which does (on the face of it) train valuable skills, but results in worse overall training outcomes due to limited skill transfer to real scenarios.

### 5.2. Worsening of Overall Net-Effects of Training

As elaborated earlier, traditional learning methods, such as video tutorials, have the benefit of being very quick and low cost to administer, while the upper end of training, e.g., live fire exercises, are exorbitantly expensive, carry a high risk of injury and require large amounts of resources. VR training should occupy the gap between these two, where cost (or complexity of administration) and utility meet.

Commercial efforts for firefighter training systems, see e.g., LUDUS (LUDUS, 2019) or NAFFCO (Sense-R, 2019) simulators, outnumber the actual evaluations of such systems with regards to training outcomes. Therefore the risk of untested systems finding their way into actual training routines of firefighters is a realistic one. As can be seen, for example, for police forces, the story of any technology implementation is a mixed one with many departments lacking *any clear success stories* (Custers, 2012) or notable increases in productivity and clearance rates (Garicano and Heaton, 2010).

The other danger with regards to training outcomes is the potential overuse of VR training systems. The overall training outcomes need to be carefully studied to find out which parts of traditional training can be supplemented, or even substituted, with VR training. Technology that should only be used as a supplementary addition to current training routines can find itself at the core of the training, creating overall worse net-effects of training outcomes. The possible cost savings might be too tempting for fire departments to refrain from overuse of VR training and thereby create worse overall training outcomes.

### 5.3. Adverse Effects of Habituation

As discussed earlier, VR training enables the high fidelity recreation of the physical and psychological conditions of the event being trained. The elicitation of arousal to match the conditions of the training task is essential to the physical fidelity of the training scenario.

Threatening situations need to be trained regularly to make sure that stress does not overwhelm firefighters in actual life threatening decision tasks (McLennan et al., 2012). While this aids the training effectiveness and preparedness, the threat of habituation could ultimately not only make training less effective, but even create a situation in which the administered training results in worse outcomes than no training at all. Habituation describes the gradual desensitization to a stimulus. This means that the repeated exposure to a stimuli can over time lead to a decreased physiological response. Repeated exposure to a physiological arousal eliciting stimuli in a VR environment can lead to habituation. Utilizing VR to habituate patients to stimuli has been used to treat phobias in patients (Mühlberger et al., 2005, 2007), but might have maladaptive side-effects for firefighter training.

A high frequency of exposure to high fidelity training environments could habituate trainees to threatening environments over time, thereby aiding the extinction of possibly life saving physiological arousal. Extinction is defined as the complete absence of a response to a stimuli. The relationship between performance and physiological arousal forms a u-shape for a broad range of behaviors that enables the highest performance once a certain amount of arousal has been reached. Too much arousal leads to a drop off in performance, whereas too little arousal hinders performance to reach optimal levels (Anderson, 1990). The threat of extinction of physiological responses could potentially lead to sub-par performance levels during the real life task.

Similarly, attention as a resource is of utmost importance for maintaining situational awareness (Endsley, 1995, 1999). While adequate training does have a positive effect on performance, training the same scenarios repeatedly can negatively impact situational awareness. The trainee becomes less responsive to the stimuli that have been trained, due to automatically activated cognitive schema, which were developed and consolidated through training (Endsley, 2000).

Research into the intensity and frequency of VR training for the target population is needed to ensure that training in high fidelity environments does accomplish desensitization to maladaptive stress responses, but not extinction of physiological stress responses for optimal performance.

### 5.4. Adverse Effects of Engagement Stimulation

Stimulating engagement is generally seen as a positive factor in simulation training of all kinds, but one has to question what this means for the physical fidelity of certain training scenarios in VR. The reality of firefighters is not always actually all that engaging. While high engagement in training exercises can lead to more extensive skill training than would be voluntary engaged in, there is arguably a gap in what the highly engaging simulation portrays and what the actual situation demands. VR training, as compared to normal simulation training, occupies a special position being naturally more engaging thanks to its' immersive properties.

Extrinsic rewards utilized when implementing game design elements in simulations, such as score systems or challenges, may have the adverse effect of over-justification. Over-justification describes the state of a trainee that has focused his effort on extrinsic rewards, while losing his desire to complete tasks for his own intrinsic feeling of achievement. This concept is well-established in psychology (Tang and Hall, 1995) and leads to decreased efforts on part of the trainees once the external reward has been removed. There is ample evidence in education research showing that poorly implemented external reward mechanisms lead to decreased intrinsic motivation of students (Chee and Wong, 2017).

Taking this concept into the domain of firefighter training, a VR environment may reach the point of becoming a too engaging environment for training with training procedures making use of extrinsically rewarding game mechanics to sustain attention during the less exciting parts of training. Once the engaging elements are removed in the real life scenario, tasks carried out may be attended to with less attention due to the removal of external rewards. This could ultimately lead to worse performance and more risk during real life execution of the trained material.

The provision of performance feedback in a simulation can also lead to overconfidence, resulting in a mismatch between perceived skill and actual real world performance. Poor performers tend to be unaware of their lack of skill (Dunning et al., 2003) and putting them in a seemingly high fidelity simulation might foster the perception of being highly skilled in the trained task. Adverse factors not salient during simulation training, such as the presence of extreme heat or general danger to life from compromised structures, might lead the trainees to obtain a skewed perception of their anticipated performance in the real world. The trainees' overconfidence can lead to potentially dangerous mistakes during deployment.

## 6. Conclusion

We have aimed to paint a holistic picture of the strengths, weaknesses, opportunities, and threats of immersive VR for the training of firefighters. VR as a training tool might seem daunting to many fire departments that rely on rigorous training to keep their staff safe, but with the low barrier to entry we argue that the technology is on its way to finding application in departments all over the world.

The large gap in costs between real life fire training and entry level VR equipment makes it possible for even small departments to have their firefighters undergo training in a large variety of scenarios in an environment with high ecological validity. The drain on resources, applicable to personnel and equipment, can be minimized and all categories of firefighters can potentially benefit from VR training without being at risk of injury or even death. Further, the control and standardization possibilities of simulation training in VR enable controlled repetition, feedback and engagement.

The advances in system engineering elaborated earlier have laid a great foundation for the future development of highly realistic environments. Using advances in multi-sensory stimulation and rendering techniques can help to increase physical fidelity to accurately portray the *feeling* of the trained scenario. Mimicking the psychological demands of the real life scenarios can further aid the mental resilience needed by the firefighting population to decrease the high rates of PTSD and suicide (Corneil et al., 1999; Antonellis and Thompson, 2012; Henderson et al., 2016).

On the other hand, there is still a large amount of work that needs to be done to mitigate weaknesses of VR technology in this domain. Without adequate user studies, using natural input methods and VR simulations highly adapted to the field, there is little knowledge in the field concerning the actual effectiveness of VR training. The technology itself also still experiences technical limitations and the issue of technology acceptance among firefighters needs to be addressed. These factors create a looming threat of uncertainty concerning the actual transfer of skills, the possible adverse mental effects of repeated VR training and ultimately the possibility of worse training outcomes due to VR training.

## Author Contributions

The analysis was conducted and written by HE. SH served in a supervisory fashion by engaging in active guidance regarding the structure of the work and provided feedback on the iterations of the manuscript prior to submission. RL served in a supervisory fashion by providing feedback on the final draft of the manuscript before submission.

### Conflict of Interest

The authors declare that the research was conducted in the absence of any commercial or financial relationships that could be construed as a potential conflict of interest.

## Footnotes

^1^https://www.gartner.com/smarterwithgartner/top-trends-in-the-gartner-hype-cycle-for-emerging-technologies-2017/

^2^https://skarredghost.com/2018/08/27/virtual-reality-is-reaching-a-mature-state-according-to-gartner/

^3^https://fireandemergency.nz/assets/Documents/Files/FENZ-Annual-Report-2017-2018.pdf

^4^https://fireandemergency.nz/assets/Documents/Files/FENZ-Annual-Report-2017-2018.pdf

^5^https://www.firetrain.com/wp-content/uploads/2017/03/Building-Description.pdf

^6^https://skarredghost.com/2018/08/27/virtual-reality-is-reaching-a-mature-state-according-to-gartner/
